# End of the ABD administration 2009-2015

**DOI:** 10.1590/abd1806-4841.2015900601

**Published:** 2015

**Authors:** Izelda Maria Carvalho Costa

On January 1st, 2009, we started our editorial adminstration of the Brazilian Annals of Dermatology (ABD). Next December 31st, 2015, it will be terminated. It has been 7 years of hard work and dedication, with the competent associate editors Andrelou Vallarelli, Renan Bonamigo and Vitor Reis.

In March 2009, after 38 years of deindexation and several efforts of the editorial team that preceeded us, headed by our colleague Prof. Bernardo Gontijo, ABD finally returned to the main and longed base PubMed/MEDLINE. This was an achievement of great importance.

Currently, a mean of more than 400 new articles per year are submitted from Brazil and from countries all around the world. In the last 7 years, over 1400 articles were published, besides 5 supplements.

The impact factor has risen steadily and, hopefully, soon the journal will no longer be B3 in Qualis/Capes, gradually reaching better positions. Moreover, we should point out that in SCIELO ranking, ABD has always been placed among the top 10 most accessed journals.

Other important achievements happened and should be mentioned, such as in 2009, when we implemented the online submission system, crucial to an indexed journal in bases of this magnitude. Also in 2009, a new section was created, the Dermatopathology section. The formatting of the magazine's cover and pages has been completely restyled since then, thus greatly improving the visual aspect of our journal. In 2010, we created the new sections Dermatology Images and Tropical Dermatology Images. It was also the year of the special commemorative stamp of 85 years of ABD. In 2011, we began to receive articles with videos so the authors could illustrate their work in a diverse and complete way. In SBD's centennial, in 2012, we edited a valuable supplement on the historical evolution of ABD. The same was thoroughly made by our team of editors and a section called "Ephemeris", present in all the issues in 2012, also collaborated with the festivities of the SBD's centennial. In 2013, ABD started to provide open access to their articles not only in SciELO, but also on PubMed Central, reaching international coverage, an important milestone for foreign readers and a great way to increase its international visibility. In 2014, the Epidemiology and Biostatistics section was created, of great importance to the scientific and academic enrichment of authors and readers. In 2015, the comemorative stamp of the 90 years of ABD was made. It was also a year of consolidation of the work developed since 2009. Today, ABD have over 120 reviewers, valuable in their opinions, and a competent editorial production, well-trained and highly dedicated to the journal.

I would like to express my deepest gratitude to all the authors who have contributed and continue to choose ABD for publication. Special thanks also to the reviewers, the editorial production (it's impossible not to mention the dedicated and sweet Vanessa Zampier) and the various boards of SBD with whom we lived and we could count on the support. Very affectionately, I thank the dear and great friends, the associate editors Andrelou Vallarelli, Renan Bonamigo and Vitor Reis. The journal would never have reached such a high level and in a relatively short time without them. I will never forget this joint and harmonious work and our beloved and nearly century-old journal, ABD - Brazilian Annals of Dermatology.

I foresee a future of glories to the official publication of the Brazilian Dermatology. It remains my hope that it is the first choice of national authors!

My sincere and best wishes to the new 2016 editors.

Prof. Dr. Izelda Maria Carvalho Costa Brasilia, spring 2015. 

